# Spatial Cognition in the Field: A New Approach Using the Smartphone’s Compass Sensors and Navigation Apps

**DOI:** 10.3390/jintelligence14010014

**Published:** 2026-01-09

**Authors:** Stefan Stieger, Selina Volsa, David Lewetz, David Willinger

**Affiliations:** Department of Psychology and Psychodynamics, Karl Landsteiner University of Health Sciences, Dr. Karl-Dorrek-Straße 30, A-3500 Krems an der Donau, Austria

**Keywords:** spatial cognition, mental rotation, spatial perception, sense of direction, celestial direction, Google maps, experience sampling method, ESMira, multi-level modeling

## Abstract

Spatial cognition refers to the mental processing, perception, and interpretation of spatial information. It is often operationalized through self-assessments like sense of direction and mental rotation ability or field-based real-world tasks like pointing to a specific building and wayfinding; however, the former and latter entail unclear ecological validity and high participant burdens, respectively. Since the advent of smartphones, this repertoire has been extended substantially through the use of sensors or apps. This study used a large longitudinal experience sampling method (ESM) in two different countries (Canada and Australia, *N* = 217) and analyzed spatial cognition both conventionally (i.e., sense of direction and speeded mental rotation test) and through new techniques like self-rated and objectively assessed daily Google Maps usage, movement patterns throughout the 14-day assessment phase (using H3 tiles for geolocation), and a Point North task. The Point North task objectively assessed deviation from the celestial direction, North, by using smartphone compass sensors. In both countries, spatial orientation was found to be associated only with the Point North task, while no significant associations were found for daily Google Maps usage (subjectively and objectively measured) and moving distance throughout the assessment phase. Although further validation is required, the Point North task shows promise as an objective, ecologically valid, and easily employable smartphone-based measure for assessing spatial cognition in real-world contexts.

## 1. Introduction

### 1.1. Spatial Cognition

Spatial cognition is the ability to process, understand, and use spatial information about the world around us to perceive, interpret, and interact with the spatial environment (Waller & Nadel, 2013). Spatial cognition is inherently interdisciplinary, with strong ties to neuroscience (e.g., the neurobiological foundations of spatial cognition; Cullen & Taube, 2017), medicine (e.g., disorders of spatial cognition in neurological diseases such as Alzheimer’s disease or stroke), and psychology (e.g., the development of spatial cognition, spatial conception, or mental rotation as a facet of intelligence).

Spatial cognition plays an important role across multiple domains of psychological functioning. (1) Spatial perception and orientation, that is, recognizing and sensing spatial relationships and distances (i.e., sense of direction). If people have high spatial perception, they are good at orienting themselves in their surroundings. (2) Mental rotation is the ability to mentally rotate representations of objects in space. In testing for this, tube figures (either round or square) are turned or flipped, and the participants must decide if they are equal. (3) When it comes to real-world applications, spatial cognition is important for navigation and wayfinding. (4) Spatial memory is a prerequisite for good wayfinding skills. Furthermore, if the participants’ spatial memory is good, they are likely to judge their direct surroundings as being familiar or recognizable.

### 1.2. Research on Spatial Cognition

Extensive research has been conducted on these aspects. It is noteworthy that early attempts to measure spatial cognition in the field were undertaken to increase ecological validity with various real-world tasks such as pointing to imagined landmarks (Bryant, 1982; Kozlowski & Bryant, 1977) or wayfinding (Cornell et al., 2003; Hegarty et al., 2006). These studies found small- to medium-sized effects, i.e., the better the self-assessed sense of direction, the smaller the “pointing error” or the better the wayfinding ability.

Previous studies have also coherently analyzed the association of mental rotation ability with sense of direction. Daily life orientation requires the ability to mentally rotate our inner presentation of our surroundings to find the right way. Interestingly, the evidence is mixed (Bryant, 1982: *r* = 0.26; Condon et al., 2015: *r* = 0.06; Hegarty et al., 2006: *r* = 0.09; Hegarty et al., 2002: *r* = 0.08) and does not indicate a strong association. It appears that sense of direction is unrelated to spatial abilities at the figural scale of space (Hegarty et al., 2002). Hegarty et al. (2006) attributed this difference to the scale of the assessed space. Mental rotation tests assess the ability on a small scale, like “perceptually examining, imagining, or mentally transforming representations of small shapes or manipulable objects, such as blocks or sheets of paper” (Hegarty et al., 2006, p. 151), whereas large-scale spaces focus on large environments (e.g., buildings, cities) that have to be experienced at many different levels (e.g., navigation, finding directions, learning, visual memory).

Familiarity with the environment is an important factor (Bryant, 1982; Cornell et al., 2003; Kozlowski & Bryant, 1977). People state that their sense of direction is better in familiar environments than in unfamiliar ones (Cornell et al., 2003) and dependent upon their experience with the environment, i.e., sense of direction also seems to have situation-specific, state-like aspects. Furthermore, in familiar environments, people with a good sense of direction perform better in pointing tasks than people with a poor sense of direction; however, this difference diminishes when both groups are placed in novel environments (Kozlowski & Bryant, 1977).

Regarding sex differences, the evidence is mixed. Although females consistently declare a lower self-rated sense of direction and mental rotation abilities than men (Bryant, 1982; Hegarty et al., 2006; Montello et al., 1999), when it comes to real-world tasks associated with spatial orientation (i.e., large-scale spatial abilities), the differences are diminished (Cornell et al., 2003) or at least reduced (Hegarty et al., 2006).

### 1.3. Spatial Cognition in the Field

Although previous research has already used real-world tasks to complement self-assessments of spatial cognition, recent methodological developments have allowed us to advance the research field further. While valuable, traditional field tasks, such as landmark pointing or wayfinding, often capture only snapshots in time; this can be resource-intensive and not fully reflective of the continuous orientation processes in natural environments. Recent methodological developments, particularly the omnipresence of smartphones, enable field research on a longitudinal basis (e.g., experience sampling method [ESM] design; Larson & Csikszentmihalyi, 1983; Mehl & Conner, 2012) in participants’ everyday life environments. They also allow the use of built-in sensors (e.g., Insel, 2017) to supplement the assessment of various facets of spatial cognition with objective data. For example, the Global Positioning System (GPS) allows us to assess geolocation. Using a longitudinal design, it is possible to analyze mobility patterns non-intrusively and objectively (e.g., mobility and depression: Laiou et al., 2022; Leaning et al., 2024). Further features, such as the compass sensor or the runtime of navigation apps (Miller, 2012), could be used to complement the picture of spatial cognition.

### 1.4. The Present Study

To draw firm conclusions, we conducted an a priori power analysis and planned the sample size to achieve at least 80% statistical power to detect small effects (i.e., effects we consider minimally relevant from a theoretical and practical point of view; H1), corresponding to a maximum Type II error probability of 20%. This increases the likelihood that future studies using a comparable design and sample size will be able to reproduce our findings, provided that the true effect size is at least small.

To avoid concentrating only on self-assessments of trait constructs (e.g., sense of direction, mental rotation), we wanted to analyze participants’ spatial cognition abilities in their everyday lives. Therefore, we applied an ESM design with high ecological validity (Larson & Csikszentmihalyi, 1983; Mehl & Conner, 2012; Stieger et al., 2023) by assessing spatial cognition concepts several times daily for two weeks.

We developed a new spatial cognition task to allow participants to point their smartphones in the cardinal direction North and assess the level of deviation from the actual North (e.g., “pointing error”). This task taps into fundamental orientation abilities, as reliance on cardinal directions is a key component of survey knowledge and efficient cognitive map use, distinct from route-following strategies (Wolbers & Hegarty, 2010). This comes with the advantage that most smartphones have built-in compass sensors. Therefore, by using the framework *ESMira* (Lewetz & Stieger, 2024) for our ESM design, we could easily and unobtrusively assess the actual direction in which participants pointed their smartphones. As this sensor had to be calibrated, we added a further objective task to determine if the sensor was calibrated correctly by letting participants point to the sun in the morning (i.e., East) and evening (i.e., West).

Furthermore, we proposed that Google Maps usage time could be an objective indicator of participants’ wayfinding abilities. In unfamiliar environments, more people with low spatial cognition abilities use Google Maps for wayfinding than those with good spatial cognition skills. To date, the objective assessment of mobility has mostly been facilitated by the use of GPS location data (e.g., Chung et al., 2021; Crane et al., 2023). However, GPS location data cannot directly assess this specific aspect of mobility or explore unfamiliar environments. With GPS data, this relationship between participants and the environment could only be inferred if large amounts of reference data were available; that is, if sufficient data were available, then a new location could be classified as familiar or unfamiliar to some degree of certainty by its proximity to existing data. If viable, an alternative indirect measure (e.g., objective Google Maps usage time) could potentially infer this information without the need for reference data, thereby enriching research on mobility.

The need to navigate is highly dependent on the familiarity of the participants’ current location (i.e., when they have good spatial memory). Therefore, we not only assessed the current location by using H3 tiles (which are more anonymous than exact GPS locations) but also asked how familiar they were with the present place. The need for navigation is likely associated with spatial ability and sense of direction. A person with a good sense of direction may quickly internalize the map of an unfamiliar environment and then navigate from memory, occasionally checking where they are. However, an individual with a poor sense of direction may need to use Google Maps in a relatively familiar setting. Some people may even have the habit of using the application during their regular commute to receive traffic updates. Therefore, this metric could potentially be influenced by other effects where the application is rarely used or conversely, frequently used even in a familiar setting. Nevertheless, the present project also aimed to analyze whether Google Maps usage time could be another useful metric.

Participants with a good sense of direction may also be more mobile, because they feel more confident while traveling. Therefore, we calculated the distance traveled during the 2-week assessment phase based on H3 tiles.

### 1.5. The Research Questions

In summary, we primarily addressed the following exploratory research questions, given the limited prior research in this area:

1. Are objective and subjective Google Maps usage patterns (as a potential measure of the wayfinding ability) associated with conventionally measured spatial cognition (sense of direction and mental rotation)?
2. Is accurate knowledge of the cardinal direction North associated with conventionally measured spatial cognition (sense of direction and mental rotation)?

Moreover, the effects of age and sex were also analyzed. Furthermore, correlations between familiarity with the environment and the distance traveled.

## 2. Methods

### 2.1. Participants

The data were collected in Canada and Australia. The two countries were selected for the following reasons: (1) English is the native language of both countries; that is, intercultural differences cannot be attributed to differences in the translation of the questionnaire; (2) the two countries are far apart; Canada and Australia are in the Northern and Southern Hemispheres, respectively. Although sunset and sunrise are in the same direction for both countries, when the smartphone is pointed at the sun (used as a “natural” validation criterion for the compass sensor), Canada is further than Australia from the equator, so slightly different angles in the validation tasks should be visible, depending on the distance to the equator. This deviation can be used as another validation criterion for *Point North* tasks.

The power analysis suggested that 80 participants per study site were necessary to reach the 80% power threshold (for detailed power considerations, see online supplement (Arend & Schäfer, 2019; Green & MacLeod, 2016)). We recruited participants using the crowd-working platform *Prolific* by selecting a sex-stratified sample (50% male, 50% female) for both subsamples (Australia, Canada). As the objective measurement of Google Maps was only possible with Android smartphones, Apple iPhone users were not invited to participate in this study. We aimed for *n* = 100 participants from each country. As 20–30% of the participants who initially agreed to participate in the longitudinal part of the study typically withdraw later, we recruited 130 participants per country (*N*_invited_ = 260).

Overall, *N* = 217 participants began the study, and 204 stayed until the end by completing the final questionnaire. As expected, the final sample was almost balanced in terms of participants’ sex (47.1% women, 48.8% men, and 4.1% other). The average age was *M* = 36.0 years (*SD* = 10.7; range: 18–83 years). Although the same number of participants was recruited from both countries, there were more Australian than Canadian citizens (53.0% and 47.0%, respectively). The dropout analysis revealed no significant demographic differences. There were no significant differences between dropouts (*n* = 15) and complete responders (*n* = 202) regarding participants’ sex (χ^2^ = 4.05, *p* = .16, φ = 0.14; slightly more males dropped out) and age (*t* = 0.50, *df* = 211, *p* = .62, Cohen *d* = 0.14; dropouts slightly younger).

### 2.2. Procedure

Participants were recruited via the online crowd-working platform Prolific. Eligible participants were required to be Canadian or Australian citizens, have English as their first language, and use an Android smartphone. Nationality was prescreened within Prolific. Within the ESMira project, we assessed nationality again to clearly distinguish between Canadian and Australian participants. Furthermore, based on the assessed geolocation (i.e., H3 tiles; see Measures section below), we were able to verify whether participants’ self-reported nationality was consistent with their current country of residence.

Furthermore, we used a sex-based quota (50% male and 50% female). The open-source software *ESMira* (Version 3.4.3; Lewetz & Stieger, 2024) was used for data collection and project administration (for details, see the online supplement). The participants were informed about the procedures and objectives by an information webpage generated by *ESMira*, and were directed to download the *ESMira* app to their personal Android smartphones to participate in the study. The data collection phase began in September 2024 and ended in early October 2024. This time of year was chosen because neither country is negatively affected by the sun’s position in the sky (i.e., high in summer, low in winter) or any other weather-related influences (e.g., winter very foggy, sun not visible due to the midnight sun relevant in northern parts of Canada).

After consenting to participate, the participants were prompted to provide their demographic details (age, sex [male, female, other], nationality [Canada, Australia, other], ZIP code), and answer questions on measures of mental health (MHC-SF, not part of this study), and their sense of direction (Santa Barbara Sense of Direction Scale: SBSoDS). Additionally, every day, the participants had to complete an end-of-day questionnaire on Google Maps usage. The notification was sent out at 8:00 p.m. local time (with a reminder after 60 min if there was no reaction; the notification deleted itself after a further 60 min without reaction). Participants could manually adapt this time point if they had conflicting daily routines (e.g., night shifts). Furthermore, the participants had to complete a time-based daily questionnaire twice per day, which entailed performing only the *Point North* task and scanning the current geolocation presented as H3 tiles. For this daily time-based questionnaire, two notifications were sent out at random times between 9:00 a.m. and 7:00 p.m. (minimum time interval between notifications = 60 min; no reminders; timeframe changeable by participants).

Furthermore, at the beginning, middle, and end of the study, further questionnaires had to be completed. A questionnaire with a mental rotation test (first day at 7 p.m.; one reminder after 60 min; on the seventh day, second administration [retest] again at 7 p.m. and one reminder; time-points not changeable by participants) and a validation task called *Point-at-sun* which was used to check if the compass sensor worked properly on the participant’s smartphone (second day at 8 a.m. in the morning and 3:30 p.m. in the evening; on the tenth day, a second administration again at 8 a.m. and 3:30 p.m.; one reminder after 30 min; time-points not changeable by participants; reminder deletion after 30 min).

At the end of the two-week assessment phase, a final cross-sectional questionnaire was completed (notification sent out at 9 a.m.; time point not adjustable by participants; reminder at the same time once a day until the questionnaire was completed), including a measure of personality (Big Five Inventory [BFI-2]; 60 items; Soto & John, 2017: not analyzed in the present study). Finally, the participants were instructed on how to receive remuneration.

The participants who completed the initial and final questionnaires, the mental rotation test (twice), and at least one time-based and end-of-day questionnaire were eligible for payment. The payment was calculated dynamically based on the overall number of filled-in questionnaires; a maximum of 9.5 British Pounds was possible (for details about the remuneration procedure, see online Supplementary Materials).

During the assessment phase, the participants could see some personal and general graphics of the collected data directly in *ESMira* (for example screenshots, see Figure S1).

### 2.3. Measures

#### 2.3.1. Initial Survey—Sense of Direction (Cross-Sectional; Once)

The SBSoDS (Hegarty et al., 2002) consists of 15 items and uses a 7-point Likert-type scale (1 = *strongly disagree*, 7 = *strongly agree*; an example item is “I very easily get lost in a new city,” “I usually let someone else do the navigational planning for long trips”). Higher scores indicate a better sense of direction.

#### 2.3.2. Mental Rotation Test (Cross-Sectional; Twice)

The stimulus library by Peters and Battista (2008; for validation, see Ganis & Kievit, 2015) was used (for an example, see screenshots of the project’s open science framework site at https://osf.io/cp5ug/ accessed on 19 December 2025). Each stimulus presented two figures comprising multiple cubes viewed from different angles. The task was to determine whether the two figures depicted the same object (i.e., only turned horizontally) or not (i.e., the objects were mirrored horizontally). We developed a short scale for this mental rotation test by randomly choosing 40 items out of the pool of 384 original items; they were balanced regarding their angles (0°, 50°, 100°, 150°; each *n* = 10) and whether they were correct or false (20 items each). In the original task, the participants had 7.5 s to answer. If a question was unanswered, the next item was shown, and the previous one was marked incorrect. As we tested in the field, we expanded this timeframe to 10 s to account for possible (unwanted and uncontrollable) influences (e.g., Internet-based loading time of picture stimuli and reduced millisecond accuracy due to parallel processes running on the smartphone).

#### 2.3.3. Point North Task (Longitudinal; Twice a Day)

In the daily questionnaire, participants had to perform a *Point North* task; that is, they were instructed to intuitively point their smartphone toward the cardinal direction North. By pressing the button “start scanning” a blue dashed circle depicted on the screen would align itself with the cardinal direction North without showing directions. When the smartphone was turned horizontally, the blue circle remained in place (because it was aligned with the North). An arrow indicated the top of the circle and was fixed. The participants were instructed to turn the smartphone horizontally until they thought the arrow pointed north (for screenshots, see Figure S2). By pressing the same button again (now labeled “stop scanning”) the cardinal direction in degrees (i.e., the deviation of the smartphones’ direction from true North assessed by the orientation sensor) is stored by *ESMira* (without showing participants).

#### 2.3.4. Geolocation—H3 (Longitudinal; Twice a Day)

On the second page of the daily questionnaire, we tried to obtain the approximate geolocation to calculate distances traveled. To avoid breaching anonymity, we did not use the longitude and latitude positions from the GPS sensor but the coarser H3 geolocation system developed by Uber. The system uses a network of hexagons that are stretched and fixed worldwide. Each hexagon has its own number and resolution range. We used resolution type 9, where each hexagon represents the size of a small district. Importantly, because the hexagons are fixed, each exact GPS location within the hexagon is always associated with the same hexagon number (e.g., 891e33289a3ffff). After pressing the scan button, the GPS location was transferred to a H3 hexagon number and displayed to the participants. The location of the hexagon could be displayed on a map using an external webpage^1^. Finally, by pressing the save button, the hexagon number was saved by *ESMira*; that is, the participants had full control over that task.

#### 2.3.5. Validation: Point-at-Sun Task (Twice a Day at the Beginning and End of the Study)

To validate the *Point North* task, a *Point-at-sun* task was developed, which was identical to the *Point North* task, with the only difference that the participants were instructed to point their smartphone to the current position of the sun in the sky; it was to be clearly visible by the participant (for exact wording, see online Supplement Figure S3). Therefore, notifications were sent out in the morning and evening, when the sun’s position was known, that is, morning east (~90°) and evening west (~270°). If the participants followed these instructions and the sensor was properly calibrated, then the sensor should show a value of approximately 90° in the morning and 270° in the evening.

#### 2.3.6. End-of-Day Questionnaire (Longitudinal; Daily)

In the end-of-day questionnaire, we assessed specific aspects of Google Maps. First, the participants were asked how long they used Google Maps on that particular day (subjective self-assessment: “For how long did you use Google Maps app on this smartphone today [in minutes]?”). Furthermore, how familiar they were with their physical environments, using a visual analog scale from 1 = *very unfamiliar* to 100 = *very familiar* (subjective self-assessment: “How familiar were you with your physical environments today?”). Finally, participants saw their objective app usage of Google Maps from the present day and the day before, which was assessed using *ESMira* (objective assessment: i.e., usage count and overall usage time, which are documented by the Android operating system by default).

### 2.4. Statistical Analyses

We used *R* (R Development Core Team, 2014) and the SPSS software (Version 30) to conduct the statistical analyses. The daily questionnaire, which was administered twice a day, included the Point North task and geolocation and assessed the current situation (Level 1; within-subject). The end-of-day questionnaire, which was administered once per day, did not assess the current situation but rather the day as a whole; it can therefore also be regarded as Level 1. The mental rotation test (based on two overall assessments), as well as the initial and final questionnaires, assessed stable traits and were therefore Level 2 (between-subject). The point-at-sun task was analyzed separately from the other questionnaires because its sole purpose was to validate the compass sensor. We calculated the distances between consecutive H3-tiles for each participant based on the chronological order of the assessment in the *Point North* task using the *H3* package^2^ in *R*. We applied Generalizability Theory Analysis (GTA; Brennan, 2002; Shrout & Lane, 2012) using the *multi-level.reliability* function in the *psych* package to analyze within-person and inter-individual reliabilities. For the analyses of Level 1 data we calculated Multi-Level Models (Nezlek, 2012; including the centering of predictors, see Enders & Tofighi, 2007) using the *lme4* (Bates et al., 2015) and *sjstat* packages (Lüdecke, 2019). For the analysis of the Level 2 data, we aggregated the Level 1 data at the person-level and calculated zero-order correlations. As some measures were skewed (e.g., usage measures of Google Maps, distance traveled), we used nonparametric measures (e.g., Table 1, Spearman correlation). All data, analysis scripts, and materials are available online at https://osf.io/cp5ug/ (accessed on 19 December 2025).

## 3. Results

Assessments came from all over Canada and Australia, mainly from west to east cities, and also from very remote areas (e.g., from Newfoundland to Chateh, an Indian reserve in northern Alberta, Canada, and Tasmania, Australia; see Figure S4). Three participants were probably on holiday because some of the data points came from Jamaica, Scotland, and Fiji.

Reliability for sense of direction and mental rotation was good for the time points and both countries (αs > 0.88; for details see Table S1). For the *Point North* task, we analyzed reliability by looking at the data on a daily basis (i.e., two assessments per day). The GTA showed that within-person reliability was rather low (*R*_c_ < 0.43; i.e., the generalizability of change—fixed time points and fixed items), suggesting that the assessments varied substantially within a day. However, the between-person reliability, by collapsing across different days of assessment, was high (*R*_kR_ > 0.89; i.e., generalizability of average time points across all items—random time effects). In general, reliability did not differ substantially across countries (for details, see Table S1).

### 3.1. Validation of Compass Sensor—Point the Smartphone Towards the Sun

To validate the accuracy of the compass sensor, we developed a simple *Point-to-sun* task by sending notifications to the participants in the morning (when the sun rises in the east at approximately 8 a.m.) and evening (when the sun sets in the west at approximately 3 p.m.). They were asked to point their smartphones towards the sun (only if the sun was visible and the task could be performed) and save the result (i.e., azimuth angle = deviation from north) using the compass sensor task. This angle was not shown to the participants. This procedure was performed at the beginning of the study and at the end to analyze the reliability of the assessments. If the sensor worked correctly, an azimuth of approximately 90° in the morning and 270° in the evening (depending on the exact latitude of the location and time of day) would be presented. Of those who regularly took part in the study, only seven participants (3.4%) did not have any data on the *Point North* or *Point-at-sun* tasks, suggesting that these participants had smartphones without a compass sensor or were unwilling to perform the task.

Participants complied with the intended procedure by performing the task in the morning or evening based on the notifications from *ESMira* (*n* = 389; see Figure S5). We categorized all measurements between 8 a.m. and 10 a.m. as morning measures and all measurements between 3 p.m. and 7 p.m. as evening measures. All other measurements (*n* = 13) were excluded as they were outside the intended assessment windows.

**Figure 1:**
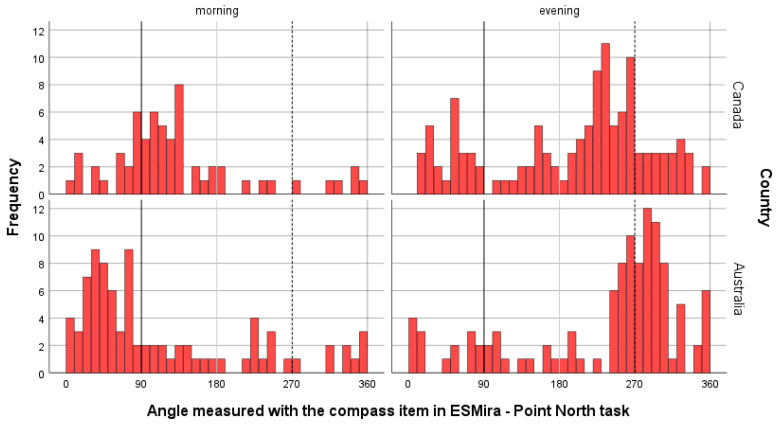
Histogram of the angle measured with the compass item (Point-at-sun task) separately for the morning and evening measurement as well as between Canada and Australia. Lines represent the position of the sun in the morning (black bold line: ~90° = east) and evening (black dashed line: ~270° = west). Angles 0° and 360° represent north and 180° south.

As shown in Figure 1, the morning azimuth measurements were predominantly close to 90° (vertical solid line) in Canada and Australia. Conversely, evening azimuth measurements were predominantly close to 270° (vertical dashed line), but with a large variation in values (for a radial view, see Figure S6). It should be noted that compass measurements can be influenced by other factors, such as magnets and strong electric fields (e.g., close to trains).

Interestingly, the mean azimuth values differed slightly between Australia and Canada. As can be seen from Figure 1, in Australia, the mean azimuth angle was slightly towards the north (<90°) for the morning measurement, which was expected because the assessment phase was from September (around the equinox) to early October, and again slightly towards the north for the afternoon (>270°). This largely depends on the exact location in Australia. Cities in the south have an azimuth of approximately 90° at sunset (e.g., Melbourne: 85° to 95° in September; for exact calculations, see https://www.timeanddate.com/ accessed on 19 December 2025). Cities in the north have azimuth values smaller than 90° at sunset (e.g., Darwin: 75° to 85° in September).

It is the opposite in Canada because it is in the Northern Hemisphere, and has a larger distance to the equator than Australia. For example, in Toronto (South Canada), the azimuth is from 95° to 97° at the end of September, that is, larger than 90°, and increases in October. It is the opposite again in the evening; that is, the azimuth angle of sunset in Toronto from the end of September onwards shifts steadily to the south (<270°) as the sun moves towards winter in the Northern Hemisphere (end of September: 266° to 270°). This trend is clearly visible in Figure 1. This indicates the validity of the task and compass sensor.

Nevertheless, we observed a substantial number of measurements that were far from 90° and 270°, which cannot be explained by the angle shift due to the distance to the equator or the month of the year. The following are possible reasons: (1) the sensor has not been properly calibrated, (2) the participants did not follow the instructions to only perform the task when the sun was clearly visible, that is, they guessed the position of the sun, (3) the assessment was influenced by other factors such as strong electric fields or magnets, and (4) the participants mixed the *Point North* and *Point-at-sun* tasks.

**Figure 2:**
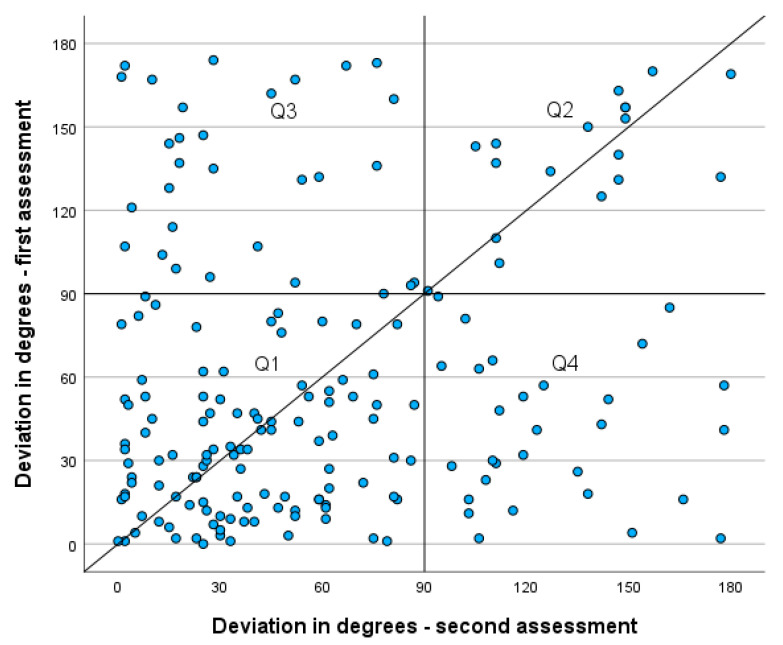
Absolute deviations from the expected azimuth angle (i.e., east, west) paired for the first assessment at the beginning of the study and second assessment at the end of the study.

To disentangle these possible effects, we analyzed the validation task in more detail. We matched the azimuth angles from the first and second assessments at the beginning and end of the study. Furthermore, we calculated the *absolute* deviation from the expected azimuth angle, which is 90° and 270° for the morning and evening measurements, respectively. The scatterplot in Figure 2 shows not only the accuracy of the first and second assessments but also how large the (absolute) deviation was from the expected angle. If the task is followed as intended and the sensor works well, we should see the data points in quadrant Q1, which was predominantly the case (57.8% of the cases). If the participants followed the instructions but the sensor was falsely calibrated (i.e., did an incorrect measurement at both the first and second measurements), then we should find data points in Q2 (9.6%). This would indicate an absolute deviation of 180°, showing that the participant actually pointed the smartphone in the *opposite* direction from the sun, not only at the first assessment but also during the second one around 10 days later. If there were other errors (e.g., participants did not follow the instructions, influence of magnetic fields on the sensor, false calibration between first and second assessments, and mixed-up tasks), we should have seen data points in Q3 and Q4 as well (in sum, 32.6%).

To test the false calibration hypothesis further, we analyzed the *Point North* task in more detail for participants who consistently pointed their smartphones in the opposite direction, where the sun was at both data points (Q2 in Figure 2). Out of those 16 participants, five (2.3%) also consistently pointed their smartphone in the opposite direction in the *Point North* task (i.e., towards the south). Eight participants clearly moved towards the north, as expected, and for three participants, no clear direction could be found. This suggests that false calibration is rare and deviations from the intended task should be attributed to alternative explanations, such as influences from other magnetic fields or misinterpretations of the used tasks.

Thus, although the Q1 values are valid, and slight deviations of values are expected according to performance location, a substantial portion seems to be influenced by other factors (Q3, Q4) or a falsely calibrated sensor (Q4; though unlikely).

### 3.2. Objective Measure of Spatial Cognition—Point the Smartphone Toward North

**Figure 3:**
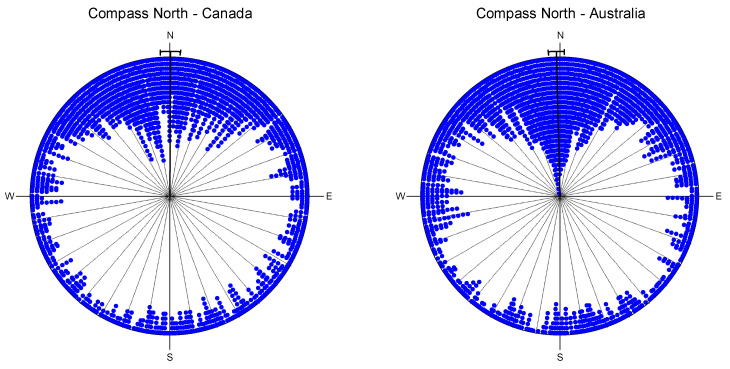
Radial histogram of all angles separated by country (Point North task). The solid bold lines represent the mean azimuth angle including a 95% confidence interval.

As shown in Figure 3, the participants demonstrated a high level of proficiency in identifying the North without reference to nationality (i.e., Canada or Australia). However, a significant variation in responses was observed, with some participants displaying responses contrary to the expected cardinal direction.

As the sense of direction and mental rotation were assessed at Level 2 (i.e., stable trait), Level 1 (longitudinal) data were aggregated at the person level. For the *Point North* task, we observed a significant correlation of absolute deviation from North with sense of direction, i.e., the better the sense of direction, the lower the absolute deviation from “true” North (−0.37, *p* < .01), and that the estimates were more accurate over time (i.e., low deviation: −0.31, *p* < .001). Conversely, we did not see a significant correlation with mental rotation ability (−0.13; see Table 1). If the small-scale vs. large-scale model of Hegarty et al. (2006) were applied, it would appear that the *Point North* task is a measure of large-scale spaces because it is associated with a large-scale sense of direction (i.e., convergent validity), but not with the small-scale mental rotation ability (i.e., discriminant validity). This pattern was also observed in Canada and Australia.

Furthermore, conventional spatial cognition assessments yielded sex differences (i.e., male participants outperformed female participants in spatial orientation, and there were no effects on mental rotation) and effects on participant age (i.e., older participants demonstrated superior spatial orientation, and there were no effects on mental rotation; see Table 1). These patterns demonstrated notable stability across countries, except for a non-significant correlation between age and spatial orientation in the Canadian data.

Interestingly, no sex or age effects were found for the subjective or objective Google Maps assessments (as a possible index of wayfinding ability) in our new measurement approaches. However, a small effect was found regarding the number of Google Maps usage counts; male subjects were found to be less frequent users of Google Maps. However, this result was unstable across countries, with the effect found only for Australian citizens but not Canadians.

Regarding *Point North* accuracy, men were only better in Canada (not Australia), and the deviation of these assessments over time was lower for men (regarding better point-North overall judgment) compared to women across both countries. Regarding the participants’ age, older participants had more accurate assessments and a lower deviation of these assessments over time. This was quite stable across countries except for a non-significant correlation with the deviation measure for Canada, although descriptively in the expected direction (see Table 1).

Familiarity with the environment was associated with all spatial cognition measures except for mental rotation. The higher the sense of directional ability, the higher the familiarity with the environment during the 14-day assessment phase. Furthermore, in highly familiar environments, Google Maps was used less often, with better knowledge of where north was. This was quite stable across countries, except for sense of direction (not significant for Australia, but the effect was nominal in the same direction), mental rotation (detrimental effects), and pointing north ability (not significant for Australia, but the effect was nominal in the same direction).

### 3.3. Reliability and Validity of the Mental Rotation Test

To test the reliability of this new short scale, we assessed the mental rotation test twice during the 14-day timeframe to analyze its stability and internal consistency (due to a reset of *ESMira’s* timetable during testing, almost all the participants completed the first test twice; therefore, we predominantly had three test administrations). Reliability was very good during both administrations (all αs > 0.87; for details see Table S1). The Intraclass Correlation Coefficient (ICC; two-way mixed-effects model, absolute agreement) over all three assessments was 0.73, suggesting acceptable stability of the score over time. Nevertheless, we also found a slight linear trend over time suggesting a learning effect (*F* = 13.8, *p* < .001, η_p_^2^ = 0.08; *M*_t1_ = 31.7, *SD*_t1_ = 6.78, *M*_t2_ = 32.7, *SD*_t2_ = 6.37, *M*_t3_ = 33.7, *SD*_t3_ = 6.15). As expected, the mental rotation score was negatively correlated with the number of missed items (*r* = −0.37, *p* < .001). The lower the mental rotation ability, the higher the probability that someone would not provide an answer within the 10 s timeframe.

To analyze the validity of the short scale, we reviewed reaction times to all items. The mean reaction time over all items was uniformly distributed, with an expected small peak at 10 s (owing to the speed of the task; see Figure S7). We only found 13 reaction times, which were extremely large. This can occur if participants switch to other apps (or are interrupted by an incoming phone call) during testing. Here, the time measurement does not stop, but automatic forwarding to the next page is not performed because *ESMira* is no longer an active application. These extremely rare cases were excluded from the data (0.05% of all reaction times). Furthermore, we analyzed very short reaction times (i.e., the clicking-through process). There were only four cases with a large click-through rate (i.e., answering within a second; 13 to 29 items had been clicked through). Due to the very low frequency (0.6%) of these cases and the subjectivity of the criteria used (<1 s), they were not excluded from further analysis.

Thus, although the testing was performed in the field (not a lab situation), where things like noise and distractions cannot be controlled for, the test scores, reliability of the re-administered test, and consistency of the test were acceptable (except for a medium learning effect over time). There were no differences in mental rotation ability between the individuals from Australia and Canada (*t* = −0.47, *p* = .642, Cohen’s *d* = −0.06). Therefore, we calculated the mean score for all test administrations for further analysis.

### 3.4. Objective and Subjective Google Maps Assessments

Participants were well able to reliably estimate their Google Maps usage on a particular day (*r* = 0.79, *p* < .001; ICC two-way mixed; absolute agreement = 0.781; for a scatter plot, see Figure S8), although we also found a mean difference in small effect size. Participants overestimated their real daily Google Maps usage only by approximately three minutes on average (*t* = 6.41, *df* = 1647, *p* < .001, Cohen *d* = 0.16, *M*_subjective_ = 16.4 min, *SD*_subjective_ = 32.22, *M*_objective_ = 13.2 min, *SD*_objective_ = 29.99).

### 3.5. Distance Traveled

Distance traveled was not significantly associated with sense of direction, mental rotation, or the accuracy of North judgments (except for a significant association with the deviation of these assessments over time, but only for Canada). However, it was significantly associated with both subjective and objective Google Maps usage. Consequently, the distance traveled appeared to be unrelated to spatial cognition, although there may be an indirect association, predicated on the hypothesis that Google Maps usage patterns could serve as an indicator of wayfinding ability.

### 3.6. Are Objective and Subjective Google Maps Usage Patterns (As a Potential Measure of Wayfinding Ability) Associated with Conventionally Measured Spatial Cognition Factors Like Sense of Direction and Mental Rotation?

As shown in Table 1, the use of Google Maps, irrespective of its assessment type (objective or subjective), exhibited no significant correlation with either sense of direction or mental rotation ability. This was consistent across Canada and Australia. Consequently, the hypothesis that Google Maps usage is indicative of wayfinding ability was not supported by these findings.

Furthermore, we calculated a multi-level regression analysis to analyze whether sense of direction or mental rotation are predictors of the (subjective, objective) patterns of Google Maps usage, even when controlling for age, sex, and familiarity with the environment. However, neither sense of direction nor mental rotation was a significant predictor (Table 2).

### 3.7. Is Accurate Knowledge of the Cardinal Direction of North Associated with Conventionally Measured Spatial Cognition (Sense of Direction, Mental Rotation)?

We calculated a multi-level regression analysis to ascertain whether the sense of direction or mental rotation are predictors of accuracy in the *Point North* task, even when controlling for age, sex, and familiarity with the environment. However, only sense of direction was a significant predictor, not mental rotation (see Table 2).

### 3.8. Follow-Up Analysis: How Stable Is the Point North Ability?

**Figure 4:**
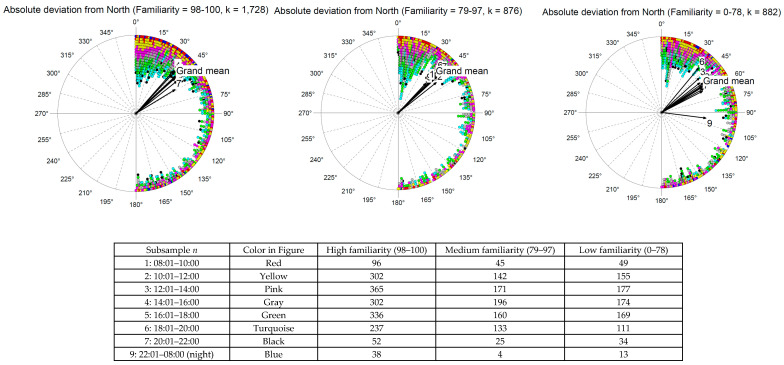
Histogram of time-of-day-specific mean values (=arrows) of the absolute deviation from North (=0/360°) based on the familiarity with the environment (0 = not familiar, 100 = very familiar). Note. Black arrows are vectors. The longer the vector, the more pronounced is the mean value, i.e., the smaller is the standard deviation, i.e., the narrower is the confidence interval.

In a follow-up analysis, we separated the data into nine different time slots of the day; that is, the time the task was performed from morning (slot 1: 8 a.m. to 10 a.m.) to night (slot 9: 10 p.m. to 8 a.m.). There were three levels of familiarity with the environment (high, medium, and low). We assumed that if people were in unfamiliar environments and performed their ratings when it was difficult to obtain additional information about the direction of North from the sun’s position (e.g., at noon, at night), they would perform poorly on the *Point North* task. As shown in Figure 4, the *absolute* deviation from North was quite stable for different time slots of the day, as was familiarity with the environment. The only substantial deviation was found for situations during night hours and low familiarity with the environment, where participants failed (Figure 4, right panel, Slot 9), although only 13 data points were available for this slot.

In summary, the participants were quite good at knowing where the cardinal direction North was. They could accomplish this independently of the time of day (i.e., in the morning and evening, this task might have been easier because the sun could be used as a reference point for North) and due to some familiarity with the environment (i.e., retrieving the cardinal direction from spatial memory because the direction North was already established in the past).

## 4. Discussion

In this study, we successfully showed that the objective field-based assessment of the ability to know the cardinal direction North aligned with the conventional self-assessment of sense of direction (*r* = −0.37; see Table 1). This association was also in line with the so-called objective *point to building* tasks used in past research (e.g., Bryant, 1982: *r* = −0.39, *p* < .05). However, we did not find strong evidence that subjective or objective Google Maps usage (as a possible marker of wayfinding ability) and mobility behavior (i.e., distance between assessment points) were associated with spatial cognition in general (i.e., sense of direction and mental rotation).

Specifically, from a *methodological* perspective, the following main results were obtained: first, only 3.4% of the participants had a smartphone with no compass sensor or were unwilling to perform the task (the latter seems unlikely because the remaining data were not affected). Second, the newly introduced *Point North* task performed well. Although false calibration was very rare (2.3% of participants), approximately 30% of the assessments seemed to be affected by either external influences (e.g., strong magnetic fields) or low commitment levels (e.g., participants not following the instructions properly). Although the within-person reliability in a single day was low, the reliability of the average time point across all items was satisfactory; that is, when using this task, spatial orientation should be longitudinally assessed by calculating a mean score to account for error variance (e.g., magnetic fields). This was also underlined by the expected correlations of the mean score with spatial orientation (*r* = −0.37, i.e., the better the self-assessed sense of direction, the lower the deviation from North in the objective *Point North* task) or participant age (*r* = −0.27, see Table 1). Third, indicators of mobility and wayfinding (e.g., Google Maps usage, distance traveled) did not correlate with a sense of direction or mental rotation ability.

From a *conceptual* perspective regarding the assessed facets of spatial cognition, we found further evidence for the small- versus large-scale hypothesis of spatial cognition (Hegarty et al., 2006). A sense of direction (i.e., large-scale spatial cognition) was not associated with mental rotation ability (i.e., small-scale spatial cognition) in either country. Our newly developed *Point North* task seemed to be a measure of large-scale spatial cognition (stable across both countries), whereas the mobility indicators (Google Maps usage patterns independent of subjective or objective assessment of traveled distance) did not correlate with either of the conventional measures of spatial cognition. Therefore, either the mobility usage patterns have nothing to do with spatial cognitions at all by measuring something different (e.g., help when traveling to unknown locations), or assess a new aspect of spatial cognitions, which needs to be analyzed in future studies.

Although there is an association with social cognition, the question remains: does the knowledge of where North lies relate to spatial cognition at all? There is evidence that birds and turtles have a magnetic compass in addition to some mammalian species, such as dogs (e.g., Hart et al., 2013; Nießner et al., 2016) and cows (Begall et al., 2008). There is also accumulating evidence that humans may still have some remnants of a magnetic sense (e.g., Chae et al., 2022; Wang et al., 2019). Although this seems a rather provocative hypothesis, the *Point North* task could assess not only spatial cognition but also the remnants of a magnetic sense. Then, this task would be separated further from objective field-based tasks like the pointing task, where participants were instructed to point to a known building (independent of any cardinal direction), or the wayfinding task, where spatial memory probably plays an important role.

### Limitations

This study has several limitations. First, the *Point North* task can be performed on its own by only focusing on the current position of the sun; a sense of direction or spatial memory is not necessary. Although the participants had been instructed to perform the task intuitively, some may have ignored that instruction. However, as can be seen in Figure 4, the deviation from north was largely independent of the time of day and familiarity of the surroundings; this is likely because finding the position of North by using the sun is easier in the morning and evening when the East and West directions can be established easily. This is more difficult at midday because the sun reaches its highest position. Future research could focus on nighttime hours to avoid such potential confounding variables.

Second, because of the field-like character of the design, it is difficult to explain why approximately 30% of the assessments in the validation task showed substantial deviations from the intended position (the sun’s position at the time of assessment). Future research should examine potential influences on the assessment of the cardinal direction North in more detail, such as (1) the influence of magnetic fields on smartphone compass sensors, (2) the context in which the assessment takes place (e.g., commuting on a train vs. being outdoors during a leisure activity), and (3) the type of environment (e.g., being in the mountains with a clear view vs. being in a large city with many tall buildings). Although the field design has clear advantages over a lab-like setting, returning to a more controlled, lab-like situation (e.g., completing the validation task in an open area with a clear view of the sun’s position, with a study administrator present) may be worth considering to better understand potential sources of deviation in the validation task.

Third, we did not control for learning or reactivity effects. Prior research has often speculated that repeatedly asking participants the same questions, providing live feedback about their data (e.g., daily Google Maps usage time), or displaying it in graphical form (e.g., familiarity with the environment over time; see Figure S1) might elicit reactivity that influences subsequent assessments. Although this could potentially threaten data quality, previous work has not found strong evidence for substantial reactivity effects (Eisele et al., 2023; Stieger et al., 2023).

Fourth, our Google Maps usage indicator was very coarse, as it was limited to daily usage time. Future research could refine this measure by assessing *how* Google Maps is used (e.g., north-up vs. egocentric view; following the navigation arrow) and how frequently the app is used.

## 5. Conclusions

This cross-national study had high ecological validity due to its field character. A sufficiently large sample was used to show that through the compass sensor of a smartphone, the newly developed *Point North* task could be used to objectively assess new facets of spatial cognition (probably rather large-scale spatial cognitions; Hegarty et al., 2006). Due to the potential influence of magnetic or electronic fields, this task should be applied in a longitudinal design to account for this error variance. Nevertheless, the task was found to be robust; that is, reliability and validity were independent of the time of day or familiarity with the environment in which the assessment took place. Indicators of mobility (i.e., subjective and objective Google Maps usage, traveled distance) did not show any substantial associations with either large-scale (e.g., sense of direction) or small-scale spatial cognition (e.g., mental rotation). Although further research on the incremental validity (i.e., usefulness among existing measures of spatial cognition) of the newly developed *Point North* task is necessary, the first results indicate that this new task could tap into new aspects of spatial cognition.

## Figures and Tables

**Table 1:** Spearman correlations between study variables.

	1	2	3	4	5	6	7	8	9	10	11
(1) Sex [1 = female, 2 = male]		0.02 <0.01	0.36 *** 0.30 **	0.06 0.08	−0.22 * 0.10	−0.29 ** −0.06	−0.10 −0.07	−0.27 ** −0.07	−0.22 * −0.06	0.15 0.09	−0.06 0.13
(2) Age [years]	<0.01		0.09 0.37 ***	0.11 0.17	−0.22 * −0.35 **	−0.13 −0.27 **	−0.12 0.15	−0.20 −0.02	−0.05 0.09	0.08 0.07	0.02 0.15
(3) Sense of direction	0.33 **	0.24 **		0.17 0.14	−0.43 *** −0.35 ***	−0.27 * −0.33 **	0.01 −0.01	−0.14 −0.12	−0.07 −0.11	0.22 * 0.18	−0.18 0.05
(4) Mental Rotation	0.07	0.14	0.15 *		−0.11 −0.17	−0.15 −0.04	−0.09 0.08	−0.14 0.07	−0.11 0.12	0.23 * −0.12	−0.06 0.11
(5) Point North: Mean absolute deviation	−0.06	−0.27 **	−0.37 **	−0.13		0.68 *** 0.69 ***	0.15 −0.13	0.22 * 0.02	0.15 −0.05	−0.26 * −0.14	0.17 −0.04
(6) Point North: SD of absolute deviation	−0.17 *	−0.19 *	−0.31 ***	−0.08	0.70 ***		0.22 * 0.02	0.16 0.04	0.14 <0.01	−0.30 ** −0.24 *	0.29 ** 0.17
(7) Google Maps: Subjective usage [min]	−0.08	0.03	<0.01	−0.01	0.10	0.46 **		0.64 *** 0.69 ***	0.76 *** 0.81 ***	−0.45 *** −0.44 ***	0.46 *** 0.45 ***
(8) Google Maps—count	−0.15 *	−0.12	−0.13	−0.04	0.10	0.09	0.67 **		0.83 *** 0.81 ***	−0.40 *** −0.40 ***	0.34 ** 0.27 *
(9) Google Maps—duration	−0.13	0.02	−0.10	0.01	0.03	0.05	0.78 **	0.83 **		−0.42 *** −0.37 ***	0.37 ** 0.38 ***
(10) Familiarity with environment	0.12	0.07	0.20 **	0.06	−0.19 **	−0.27 ***	−0.44 ***	−0.40 **	−0.39 **		−0.34 ** −0.38 ***
(11) Distance traveled	0.03	0.09	−0.07	0.03	0.06	0.23 **	0.46 ***	0.32 **	0.37 **	−0.36 **	

*Note*. Demographics in gray, Spatial cognition—conventional measures in light red, Spatial cognition—new approaches in light green, Spatial cognition—additional spatial variables. Sex = *other* has been excluded (*n* = 9). Point North: Mean deviation = Mean absolute deviation from North [degrees], Point North: SD of deviation = Standard absolute deviation from North [degrees], Distance traveled = Mean distance between two consecutive assessment points [km], Google Maps—count: Objective mean usage [count per day], Google Maps—duration: Objective mean usage [minutes per day]. Above diagonal country-specific results. First line entry = Canada (*N* = 84–98), second-line entry = Australia (*N* = 88–110). * *p* < .05, ** *p* < .01, *** *p* < .001.

**Table 2:** Results of the multi-level model.

	Google Maps Usage			*Point North* Task
	Subjective	Objective Count	Objective Duration	Absolute Deviation
	Standardized *B* (=β)			
Sense of direction	0.08	0.08	0.09	−0.18 ***
Mental rotation	<0.01	<0.01	0.03	−0.09
Age	>−0.01	−0.05	0.02	−0.12 **
Sex	−0.02	−0.14	0.01	0.11
Familiarity	−0.33 ***	−0.16 ***	−0.35 ***	−0.05 **

*Note*. Familiarity = Familiarity with the environment on a particular day. ** *p* < .01, *** *p* < .001.

## Data Availability

The raw data and materials can be found at https://osf.io/cp5ug/ (accessed on 19 December 2025). The code used for analyses is available in the OSF repository, https://osf.io/cp5ug/ (accessed on 19 December 2025). The used software *ESMira* is an open-source project which can be found at https://github.com/KL-Psychological-Methodology/ESMira (accessed on 19 December 2025).

## Notes

1 https://wolf-h3-viewer.glitch.me/?h3=891e33289a3ffff (accesssed on 19 December 2025).

2 https://h3geo.org/ (accesssed on 19 December 2025).

## Supplementary Materials

The following supporting information can be downloaded at: https://www.mdpi.com/article/10.3390/jintelligence14010014/s1.
