# Supplementary material for: Spatial Cognition in the Field: A New Approach Using the Smartphone’s Compass Sensors and Navigation Apps
Source: J Intell. 2026 Jan 9;14(1):14. doi: 10.3390/jintelligence14010014 (PMC12842908; doi:10.3390/jintelligence14010014)
Supplement: Supplementary file 1 [file jintelligence-14-00014-s001.zip › jintelligence-3919676-supplementary.pdf]

Supplement

Spatial Cognition in the Field: A New Approach Using the Smartphone's Compass Sensors and Navigation Apps

Supplementary methods

Power considerations

We used an elaborated approach to simulate power in multi-level designs by using the R-package *simr* (Green & MacLeod, 2016). We used the following assumptions: α = 5%, intraclass correlation ICC = .30, number of retests = 28 (two assessments per day, duration 14 days), number of participants = 100 to start with. Furthermore, to be cautious, we assumed small effect sizes for the standardised effects at Level 1 and 2 and cross-level interactions (i.e., .1), and a random slope for Level 1 effects of .01 (small effect; for recommendations, see Arend & Schäfer, 2019). Using 1,000 simulations and testing several combinations between number of participants and number of retests (assuming a dropout during data collection phase), we reach a power of 99.80% (95% CI = 99.28, 99.98) for 100 participants and 28 retests, and power of 80.60% (95% CI = 78.01, 83.01) for 80 participants and 14 retests (assuming 50% non-response to the daily questionnaires; for all calculated combinations, see https://osf.io/cp5ug/). Therefore, we aimed for at least 80 participants for each study site.

ESMira

ESMira (Lewetz & Stieger, 2024) is especially designed for longitudinal scientific studies (Experience Sampling Method; ESM) by offering a wide repertoire of functions and possibilities for scientific data collection (e.g., presentation and consent of the informed consent form, data encryption, data security, anonymous chat function, graphical feedback, anonymity through randomly generated codes, anonymous reward option). ESMira is available for both Android and iOS operating systems (https://github.com/KL-Psychological-Methodology/ESMira).

Timepoints to complete the surveys were predefined by the authors, and participants received a notification (i.e., signal, in-app reminder) through ESMira on their personal smartphone, asking them to complete a particular questionnaire. After selecting that notification message, the respective questionnaire opened automatically.

Communication with participants

Due to the anonymous character of the study, it was important to give participants support whenever a problem occurred. We used the built-in chat function of ESMira to allow participants to send the study administrators questions anonymously. Furthermore, study administrators could send specific (or also groups of) participants a message, if, for example, the final questionnaire was not completed yet.

Personalised graphics

During the study, participants could view general as well as personalised statistics in a graphical format directly in their ESMira app. Almost all participants (*n* = 184, 84.8%) used this option at least once (*M* = 7.2 times, *SD* = 11.24, Median = 3, range 1 to 78).

Remuneration procedure by ESMira

Participants were instructed to press the reward button in ESMira when the study was over. ESMira then checked if they were eligible for the reward. If yes, ESMira presented an individual anonymous reward code, which had to be sent to the study administrators via Prolific. If they were not eligible, ESMira stated which questionnaires were missing. In the server-based admin-tool of ESMira, the reward code could be verified for validity (in order to avoid misuse). This procedure guaranteed that the anonymity of the participant was not breached by using the ESMira-specific user ID or participant’s email address instead of the reward code, and that the codes were valid (for more details, see Lewetz & Stieger, 2024).

Figure S1.

Graphical Feedback in ESMira

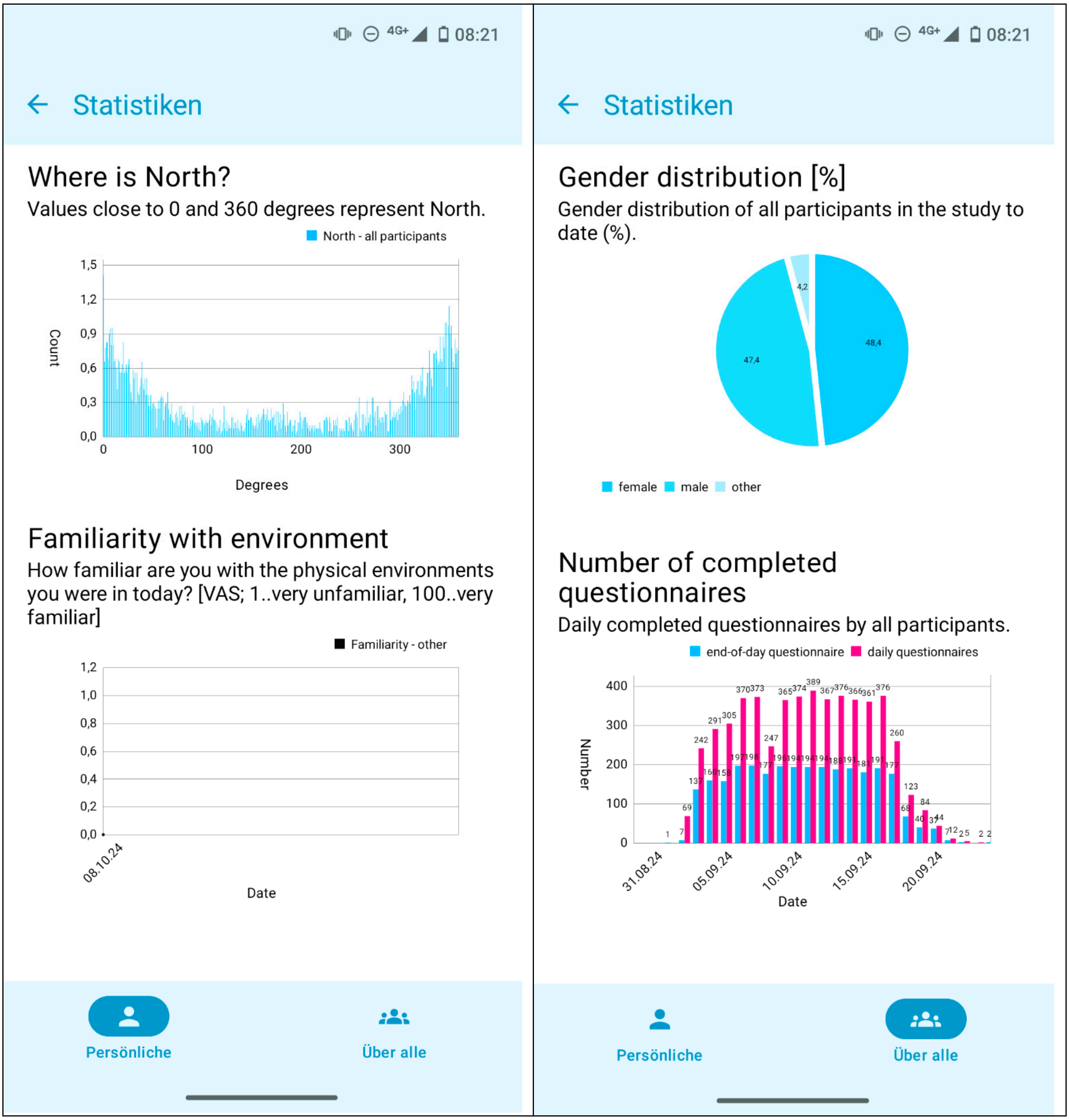

Figure S2.

Point North Task (left panel) and the H3-Tile Geolocation Page (right panel)

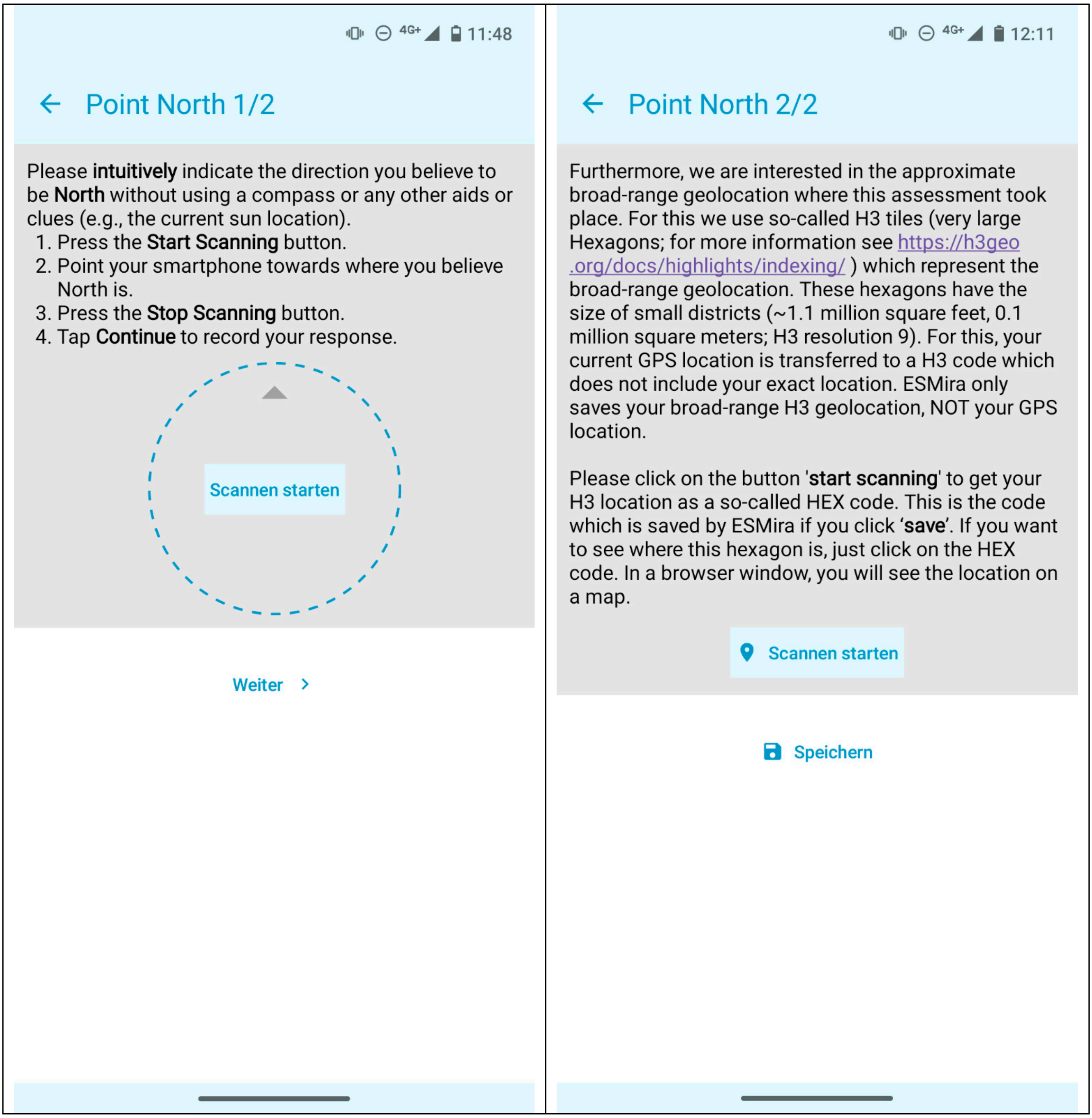

Figure S3.

Screenshot of the Point-at-sun *task*

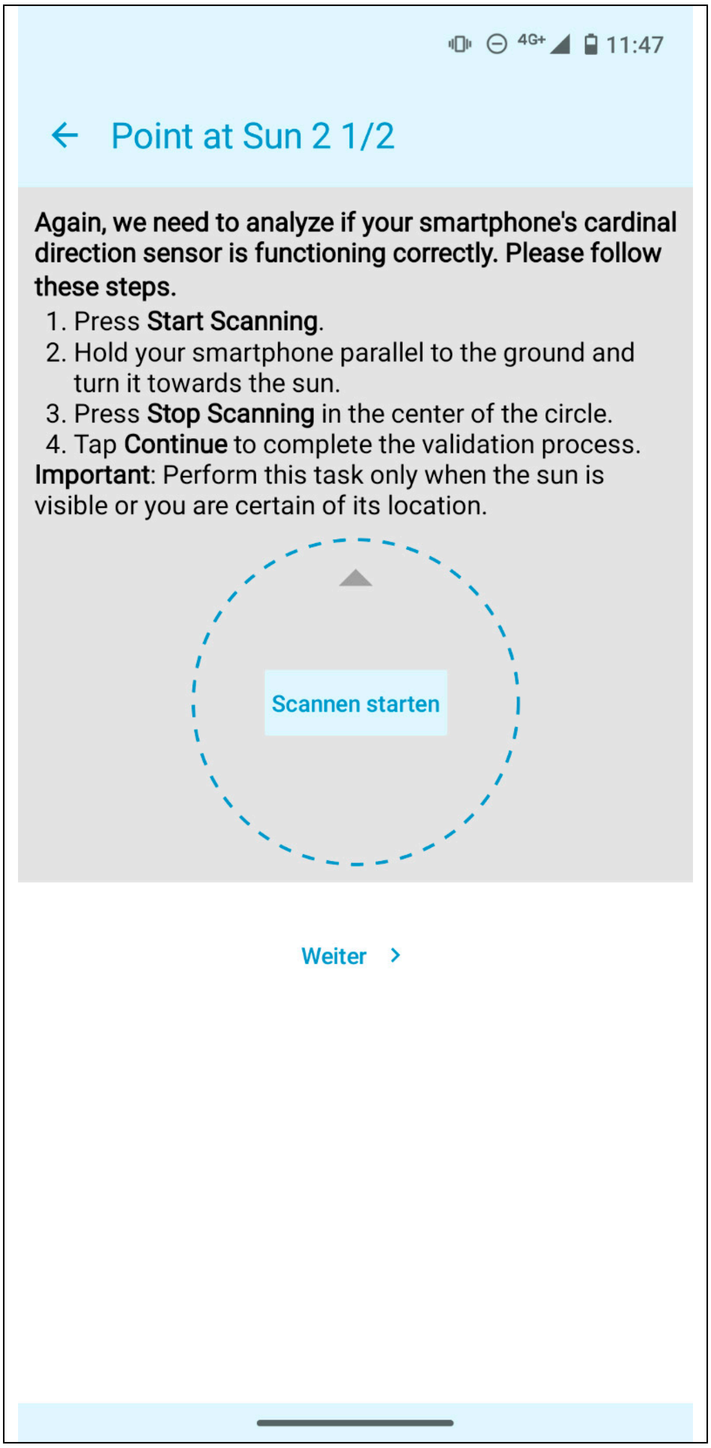

Figure S4.

Geolocations (H3 tiles, resolution = 4 out of 9 for better visibility) of all Point North *task assessments (*k *= 3,583). Note. Datapoints from Jamaica, Scotland, and Fiji are not shown.*

Canada

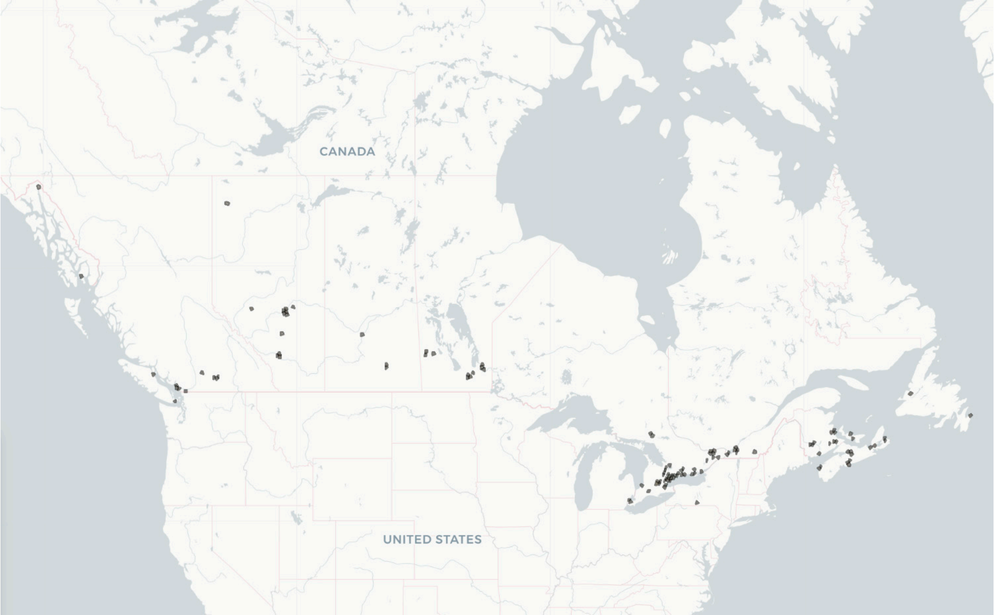

Australia

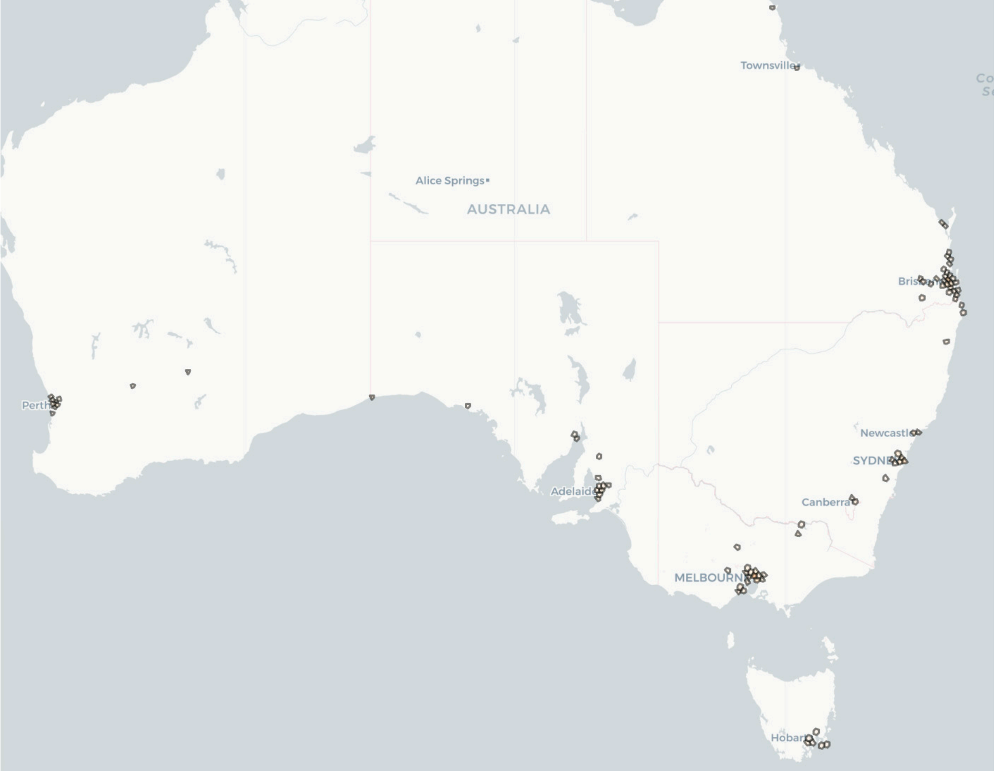

Figure S5.

Histogram of the time point, participants did the 'point-to-sun' task.

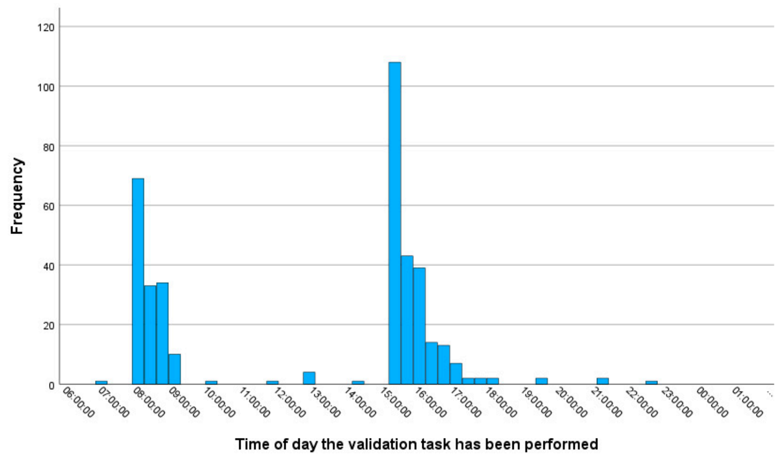

Figure S6.

Radial histogram of all angles separated by timepoint (morning, evening). Solid bold line represents the mean including a 95% confidence interval.

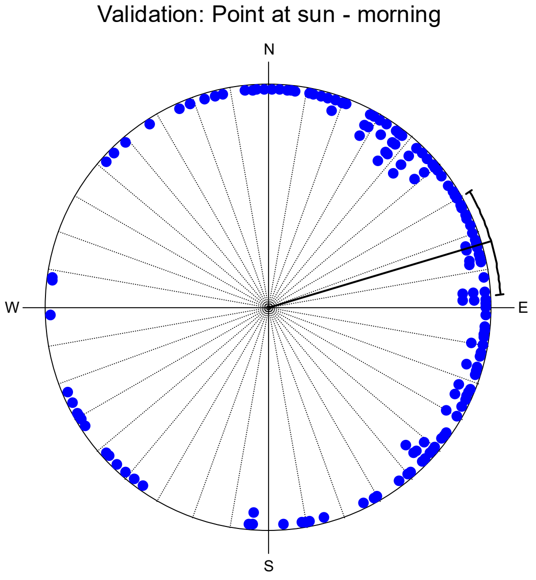

All datapoints for morning assessment (notification sent 8 a.m. local time; time of doing the task: range 8:01 to 9:17 a.m.)

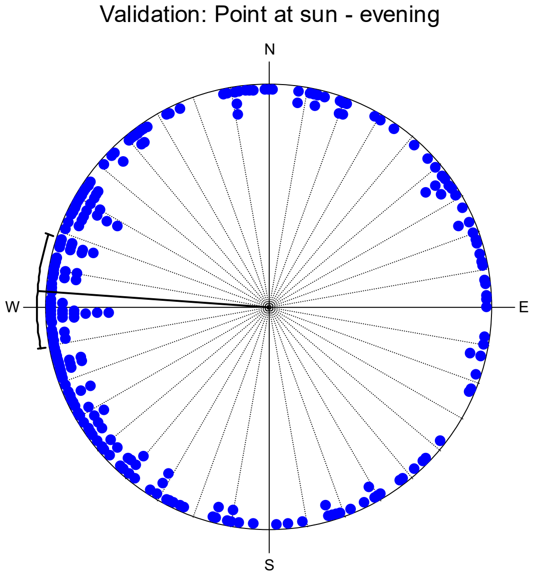

All datapoints for evening assessment (notification sent 3 p.m. local time; time of doing the task: range 3:17 to 6:15 p.m.)

Figure S7.

Histogram of the mean reaction times of the Mental Rotation test.

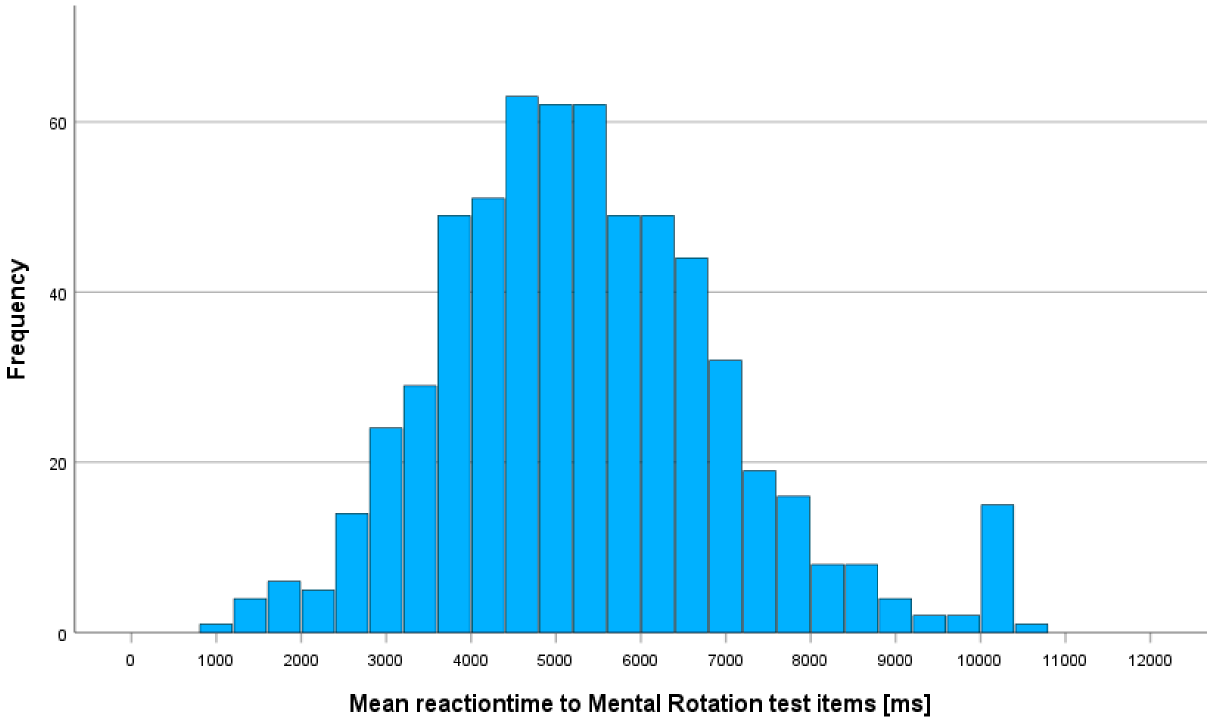

Figure S8.

Scatterplot for the subjective and objective daily Google Maps usage in minutes (dotted line: perfect match, solid line: actual match = regression line)

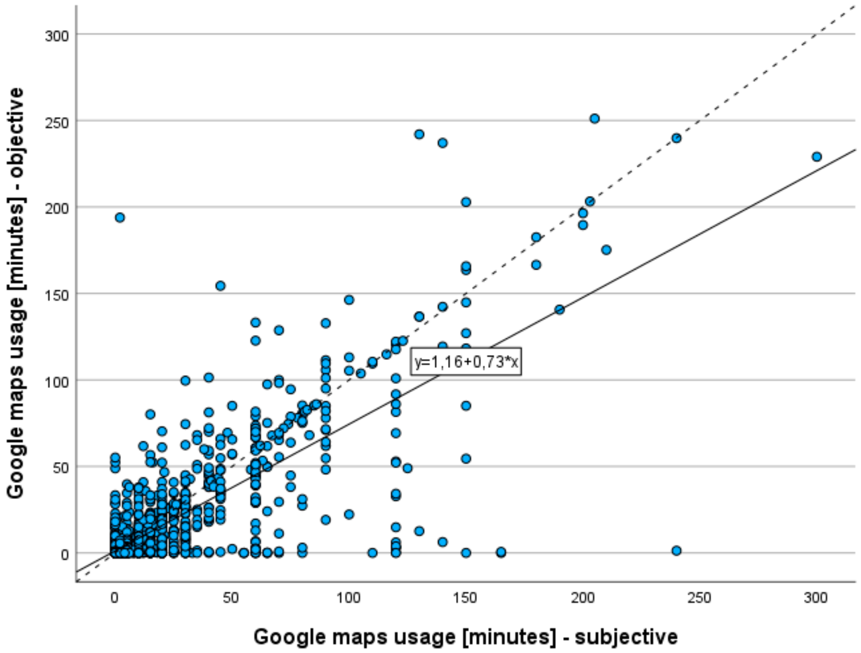

Table S1.

Reliability of all between- and within-subject measures separately for Canada and Australia

	Canada	Australia
	Cronbach α	
Santa Barbara Sense of Direction Scale - SBSoDS	.907	.917
Mental Rotation (t_1)	.893	.889
Mental Rotation – Retest (t_2)	.881	.868
	Generalizability Theory Analysis	
Deviation from North – R_c	.43	.21
Deviation from North – R_{kR}	.89	.93

Note. R_c = generalizability of change—fixed time points and fixed items; R_{kR} = generalisability of average time points across all items—random time effects.

References

Arend, M. G., & Schäfer, T. (2019). Statistical power in two-level models: A tutorial based on Monte Carlo simulation. *Psychological Methods, 24*(1), 1–19.

Green, P., & MacLeod, C. J. (2016). SIMR: An R package for power analysis of generalized linear mixed models by simulation. *Methods in Ecology and Evolution, 7*, 493–498.

Lewetz, D., & Stieger, S. (2024). ESMira: A decentralized open-source application for collecting experience sampling data. *Behavior Research Methods, 56*, 4421–4434. http://doi.org/10.3758/s13428-023-02194-2
